# Association between dietary magnesium intake and muscle mass among hypertensive population: evidence from the National Health and Nutrition Examination Survey

**DOI:** 10.1186/s12937-024-00940-6

**Published:** 2024-03-21

**Authors:** Qin Wang, Keyi Si, Xiaohong Xing, Xiaofei Ye, Ziyu Liu, Jing Chen, Xiaojing Tang

**Affiliations:** 1 Department of Health Management, Naval Medical University, Shanghai, China; 2 Department of Military Health Statistics, Naval Medical University, Shanghai, China; 3 Department of Nephrology, Shanghai Changzheng Hospital, Naval Military Medical University, No. 415 Fengyang Road, Shanghai, 200003 China; 4 Department of Nutrition and Food Hygiene, School of Public Health, Fudan University, Shanghai, China

**Keywords:** Hypertension, Magnesium intake, Muscle mass

## Abstract

**Background:**

Magnesium is critical for musculoskeletal health. Hypertensive patients are at high risk for magnesium deficiency and muscle loss. This study aimed to explore the association between magnesium intake and muscle mass in patients with hypertension.

**Methods:**

In this population-based cross-sectional study, 10,279 U.S. hypertensive adults aged 20 years or older were derived from the National Health and Nutrition Examination Survey in 1999–2006 and 2011–2018. Magnesium (Mg) intake from diet and supplements was assessed using 24-hour diet recalls. Muscle mass was evaluated by appendicular skeletal muscle mass index (ASMI, total ASM in kilograms [kg] divided by square of height in meters [m^2^]). The association of Mg intake with ASMI was estimated using weighted multivariable-adjusted linear regression models and restricted cubic splines.

**Results:**

Dose-response analyses showed a positive linear correlation between dietary Mg intake and ASMI. Every additional 100 mg/day in dietary Mg was associated with 0.04 kg/m^2^ (95% confidence interval [CI] 0.02–0.06 kg/m^2^) higher ASMI. The ASMI in participants who met the recommended dietary allowance (RDA) for dietary Mg was 0.10 kg/m^2^ (95% CI 0.04–0.16 kg/m^2^) higher than those whose dietary Mg was below estimated average requirement (EAR). However, the relationship of Mg intake from supplements with ASMI was not identified.

**Conclusion:**

Higher level of dietary Mg intake rather than Mg supplements was associated with more muscle mass in U.S. adults with hypertension, which highlights the importance of meeting the recommended levels for dietary Mg intake.

**Supplementary Information:**

The online version contains supplementary material available at 10.1186/s12937-024-00940-6.

## Introduction

The prevalence of hypertension was 24.0% in U.S. adults aged 18 ∼ 44 years, 58.0% in those aged 45 ∼ 64 years and 75.4% in those aged 65 ∼ 74 years [1]. Muscle loss is a natural result of aging with a rate of approximately 3–8% per decade after the age of 30 and the degeneration might be more severe in patients with hypertension [2]. It is reported that low muscle mass was more prevalent in hypertensive patients when compared with normotensive counterparts (41.9% vs. 13.2%) [3]. Previous studies have illustrated a vicious cycle between arterial stiffness, an indicator of hypertension, and muscle loss [4]. Increased arterial stiffness might result in reduced blood flow to the limbs, and subsequently lead to decreased muscle mass [5, 6]. On the other hand, reduced muscle mass is related to low physical activity, insulin resistance [7], chronic inflammatory state, and oxidative stress [8], which are all significant contributors to increased arterial stiffness. Moreover, comorbid hypertension and low skeletal muscle mass may increase the risk of negative health outcomes, including fracture and falling incidents [9], cognitive impairment [10], and albuminuria [11]. Given the adverse consequences of muscle loss in hypertension, its prevention strategies are of great significance and necessity.

Nutritional therapy may be a promising method in mitigating muscle loss [12, 13]. It is recognized that various micronutrients, such as magnesium (Mg), are critical for musculoskeletal health. The human body contains 760 mg of Mg at birth [14]. A dynamic balance of approximately 25 g Mg is then achieved by the interplay between intestinal absorption and renal excretion in an adult body [15]. About 27% of Mg is contained in the skeletal muscles where it is important for muscle contraction and relaxation [16, 17]. Mg can be obtained from foods, beverages, supplements, fortified food, and some medications such as antacids containing hydrotalcite. Whole grain, seeds, nuts, legumes, and cocoa are considered good sources of Mg [14]. Epidemiological studies have shown that higher dietary Mg intakes were associated with greater skeletal muscle mass and muscle strength in older adults [18–20]. However, whether the association applies to hypertensive patients who are at high risk of muscle loss is unknown. Besides, whether the sources of Mg modify the association is rarely discussed.

Thus, this study aimed to identify the relationship between Mg intake (diet and supplement) and skeletal muscle mass among population with hypertension based on a representative sample from the National Health and Nutrition Examination Survey (NHANES).

## Materials and methods

### Data source and study population

**Fig. 1:**
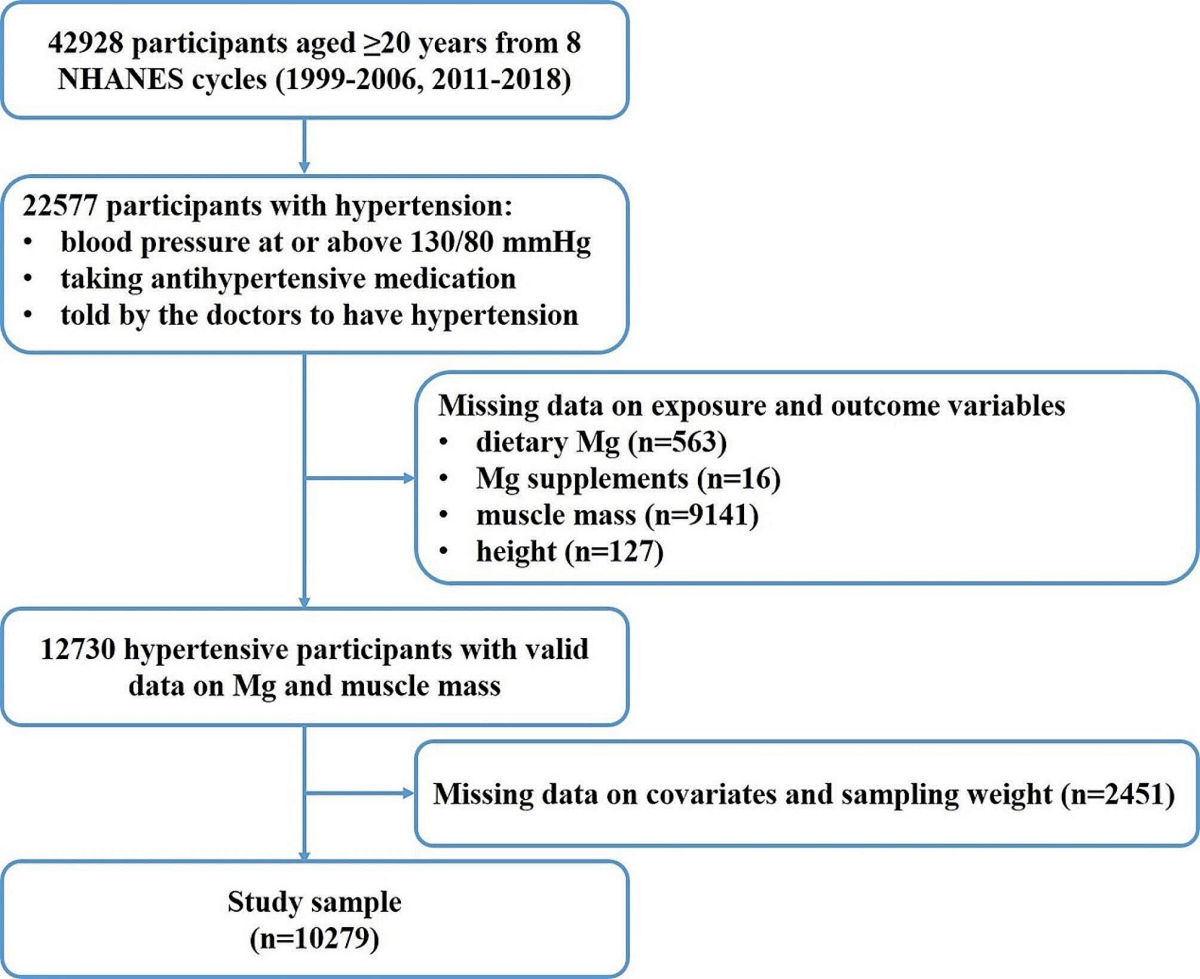
Inclusion and exclusion of participants. *Abbreviations* Mg, magnesium; NHANES, National Health and Nutrition Examination Survey

The NHANES is a large ongoing program designed to assess people’s health and nutritional status in the U.S. by examining a nationally representative sample of about 5,000 people each year since 1999. The survey included health interviews conducted in-person and by telephone, medical and physical measurements in specially designed mobile examination centers (MEC), and laboratory tests. In the current study, 22,577 adults aged 20^+^ years with pre-existing hypertension were first extracted from the cycles 1999–2006 and 2011–2018, when muscle mass was measured annually. After excluding participants with missing data on Mg intake (*n* = 579), including diet and supplements, muscle mass and body height (*n* = 9,268), and the covariates and sampling weight (*n* = 2,451), 10,279 participants were left in our final analyses (Fig. 1). Informed consent was provided by all participants.

### Definition of hypertension

Three and sometimes four blood pressure determinations (systolic and diastolic) were taken by trained physicians in the MEC and during home examinations using a mercury sphygmomanometer (W.A.Baum Co., Copiague, NY, USA) after the participants remained in seating position for at least 5 min. Participants who met one of the following criteria were diagnosed with hypertension: (1) participants who were told by the doctors to have hypertension; (2) the mean value of diastolic blood pressure (DBP) ≥ 80 mmHg or the mean value of systolic blood pressure (SBP) ≥ 130 mmHg; (3) use of hypertension medication by self-report [21]. Mean blood pressure was calculated based on NHANES’s recommendation (https://wwwn.cdc.gov/nchs/nhanes/2001-2002/BPX_B.htm).

### Exposure and outcome measures

Total Mg intake was calculated as the sum of daily Mg intake from diet and supplements. Dietary Mg intake was calculated by the mean value of two 24-hour dietary recalls—one in-person in the MEC, the other by telephone 3–10 days later. First, the amount of all foods and beverages except drinking water that had been consumed during the previous 24 h was quantified using food-specific units, abstract food models, special charts, and other measurement aids. Then, dietary intake of Mg was calculated based on the amount of each type of food/beverage and the corresponding nutrient value using the U.S. Department of Agriculture’s Food and Nutrient Database for Dietary Studies [22]. The use of supplements (not limited to Mg) in the past 30 days was first inquired during home examinations. Then, two 24-hour recalls on dietary supplements (including non-prescription antacids) were conducted among supplement users in the MEC and by telephone. Mean Mg intake from supplements was calculated by combining the frequency with the product information on ingredient, amount of ingredient per serving, and ingredient unit. If data were available for only one recall, the single value was used instead of an average.

The outcome variable was muscle mass defined by appendicular skeletal muscle mass index (ASMI), which is calculated by the ratio of total ASM (kg) to the square of height (m^2^). ASM was measured by dual-energy X-ray absorptiometry (DXA) whole-body scans and standing height by a wall-mounted digital stadiometer. The cut-off points for low muscle mass were ASMI less than 7 kg/m^2^ for men and 5.5 kg/m^2^ for women [23]. Due to gender differences in skeletal muscle mass, ASMI was cut into gender-specific tertiles.

### Potential confounding variables

The potential confounding variables in this study were determined based on previous studies [24–26], including age (< 45years, 45 ∼ 65years, ≥ 65years), gender, race (Mexican Americans, other Hispanic, non-Hispanic White, non-Hispanic Black, and other race), poverty income ratio (PIR, < 1, 1–3, and ≥ 3), body mass index (BMI), physical activity (less than moderate, moderate, vigorous), smoking status (never, former, and current), drinking status (never, former, and current), total energy intake, protein intake, diabetes, heart disease, chronic kidney disease, total cholesterol, serum albumin, C-reactive protein (CRP), and muscle-related medications.

Diagnoses of diabetes and heart disease were based on an affirmative response to the question “Has a doctor or other health professional ever told you that you had diabetes, coronary heart disease, congestive heart failure, heart attack, or angina?”. Participants were also considered diabetic if they were treated for diabetes, or had a hemoglobin A1c (HbA1c) of 6.5% or more. Participants with estimated glomerular filtration rate (eGFR) < 60 mL/min/1.73m^2^ calculated by chronic kidney disease Epidemiology Collaboration equation [27] or randomized urinary albumin/creatinine ratio (ACR) > 30 mg/g were considered patients with chronic kidney disease. Total energy intake and protein intake were determined by the mean value of the two 24-hour recall data. Self-reported prescription data was used to determine if the patient was taking muscle-related medications including statins, sulfonylureas, and glycinates.

### Statistical analyses

Given the complex probability sampling design of NHANES, all the statistical analyses took weights into account. Continuous variables were presented as mean ± standard deviation or median with an interquartile range. Categorical variables were presented as number (weighted percentage). Statistical differences were determined using a weighted *t* test for continuous variables, while a weighted χ^2^ test was used for categorical variables.

To investigate the potential non-linear relationship between Mg intake and ASMI, a restricted cubic spline (RCS) analysis was conducted, with adjustment for age, gender, race, PIR, BMI, physical activity, smoking status, drinking status, total energy intake, protein intake, diabetes, heart disease, and chronic kidney disease, total cholesterol, serum albumin, CRP, and use of muscle-related medications. If the relationship was linear, *β* (95% confidence interval, [CI]) of ASMI for every additional 100 mg/day Mg intake was calculated using weighted multivariable linear regression models. Linear regression assumptions (normality, equal variance, linearity, and independence) were tested and no violation was observed. Mg intake was also divided into three groups according to the estimated average requirement (EAR) and recommended dietary allowance (RDA) by Institute of Medicine (US) Standing Committee on the Scientific Evaluation of Dietary Reference Intakes (Supplementary Table S1): < EAR (reference), EAR–<RDA, and ≥ RDA [28]. To explore whether the association was modified by age, gender, PIR, BMI, physical activity, diabetes, heart disease, chronic kidney disease, serum albumin, CRP, and use of muscle-related medications, we conducted interaction analyses and stratified analyses. Since protein-energy wasting could influence muscle atrophy [29, 30], we also considered low energy intake (energy intake < 25 kcal/kg/day) and low protein intake (< 0.6 g/kg/day) in the stratified analyses.

Moreover, we did several sensitivity analyses to test the robustness of our results. The main analyses were repeated in participants who met the criteria of SBP ≥ 140 mmHg and /or DBP ≥ 90 mmHg and in participants without hypertension history.

The software packages R (version 4.2.2, The R Foundation) and Free Statistics software version 1.7 were used to perform all statistical analyses. A two-sided *P*-value < 0.05 indicated significance for all analyses.

## Results

### Baseline characteristics

About 52.0% of the hypertensive participants did not meet the EAR for Mg intake and 31.2% reported the use of Mg supplements. The prevalence of low muscle mass was 9.2% in the whole participants. The characteristics of hypertensive participants according to ASMI tertiles are shown in Table 1. More participants in the higher tertile were male and Mexican American. Those in the higher tertile group were younger, had a higher intake of protein and energy, were more likely to be obese and diabetic, but less likely to smoke, take Mg supplements, or have heart diseases.

**Table 1:** Characteristics of participants stratified by gender specific tertiles of ASMI^a,b^

	ASMI (kg/m^2^)				
	All: 8.1 ± 1.7 (*n* = 10,279)	Tertile 1: 6.5 ± 1.0 (*n* = 3427)	Tertile 2: 8.0 ± 0.9 (*n* = 3427)	Tertile 3: 9.8 ± 1.3 (*n* = 3425)	P value
**Age (years), n (%)**					*< 0.001*
< 45	3469 (37.3)	747 (26.3)	1113 (36.8)	1609 (49.0)	
45–65	4547 (48.4)	1372 (48.7)	1642 (50.5)	1533 (45.8)	
≥ 65	2263 (14.3)	1308 (25.1)	672 (12.7)	283 (5.2)	
**Male, n(%)**	5627 (55.4)	1876(51.6)	1876 (56.9)	1875 (57.5)	*0.002*
**Race, n (%)**					*< 0.001*
Mexican American	1775 (6.7)	635 (5.2)	657 (7.0)	483 (7.9)	
Non-Hispanic White	4830 (70.2)	1966 (77.4)	1630 (72.9)	1234 (60.3)	
Other Hispanic	524 (4.5)	143 (3.8)	198 (5.0)	183 (4.6)	
Non-Hispanic Black	2368 (12.2)	359 (5.2)	684 (9.9)	1325 (21.5)	
Other race	782 (6.5)	324 (8.4)	258 (5.2)	200 (5.8)	
**PIR, n (%)**					*0.08*
< 1	1744 (12.2)	570 (12.4)	573 (11.4)	601 (13.0)	
1–3	4397 (36.5)	1525 (37.7)	1406 (34.5)	1466 (37.5)	
≥ 3	4138 (51.2)	1332 (49.9)	1448 (54.1)	1358 (49.5)	
**BMI (kg/m** ^**2**^ **), n (%)**					*< 0.001*
< 30	5754 (56.2)	3243 (94.9)	2082 (60.9)	429 (12.2)	
≥ 30	4525 (43.8)	184 (5.1)	1345 (39.1)	2996 (87.8)	
**Physical activity, n (%)**					*0.30*
Less than moderate	4611 (40.3)	1554 (41.3)	1490 (38.2)	1567 (41.4)	
Moderate	3241 (35.3)	1077 (35.2)	1104 (36.2)	1060 (34.4)	
Vigorous	2427 (24.4)	796 (23.5)	833 (25.6)	798 (24.1)	
**Smoking, n (%)**					*< 0.001*
Never	5209 (49.7)	1548 (43.6)	1749 (50.0)	1912 (55.5)	
Former	2809 (27.5)	1037 (28.5)	932 (27.1)	840 (26.8)	
Current	2261 (22.8)	842 (27.8)	746 (22.9)	673 (17.7)	
**Drinking, n (%)**					*0.002*
Never	1288 (10.2)	476 (11.5)	402 (9.3)	410 (9.9)	
Former	1876 (15.9)	599 (14.8)	602 (14.7)	675 (18.3)	
Current	7115 (73.9)	2352 (73.7)	2423 (76.0)	2340 (71.8)	
**Diabetes, n (%)**	1890 (14.1)	499 (9.7)	571 (12.3)	820 (20.3)	*< 0.001*
**Heart disease, n (%)**	1015 (8.0)	436 (10.1)	320 (8.0)	259 (5.9)	*< 0.001*
**Chronic kidney disease, n (%)**	2014 (14.6)	768 (16.0)	585 (12.6)	661 (15.2)	*0.005*
**CRP (mg/dL), median (IQR)**	0.3 (0.1, 1.0)	0.2 (0.1, 0.6)	0.3 (0.1, 0.9)	0.6 (0.2, 1.8)	*< 0.001*
**Total cholesterol (mmol/L)**	205.1 ± 43.0	207.7 ± 41.8	207.3 ± 43.7	200.3 ± 42.9	*< 0.001*
**Serum albumin (g/L)**	43.2 ± 3.4	43.6 ± 3.4	43.5 ± 3.2	42.5 ± 3.4	*< 0.001*
**Energy intake (kcal/day)**	2180.7 ± 943.1	2047.1 ± 903.2	2214.7 ± 951.8	2279.9 ± 958.2	*< 0.001*
**Protein intake (g/day)**	84.1 ± 39.3	76.5 ± 35.4	85.5 ± 39.9	90.2 ± 41.1	*< 0.001*
**Total Mg intake (mg/day)**	331.7 ± 184.7	328.5 ± 188.1	335.7 ± 186.8	330.9 ± 178.8	*0.14*
**Total Mg intake according to U.S. dietary guideline, n(%)**					*0.30*
<EAR	5874 (52.0)	1969 (52.4)	1914 (50.2)	1991 (53.5)	
EAR-<RDA	1675 (17.6)	540 (17.5)	584 (18.7)	551 (16.4)	
≥ RDA	2730 (30.5)	918 (30.1)	929 (31.1)	883 (30.1)	
**Dietary Mg intake (mg/day)**	295.8 (139.0)	284.0 (130.2)	302.3 (141.8)	300.9 (143.9)	*< 0.001*
**Dietary Mg intake according to U.S. dietary guideline, n(%)**					*0.009*
<EAR	6736 (61.0)	2355 (64.6)	2178 (58.6)	2203 (59.8)	
EAR-<RDA	1552 (16.5)	466 (14.9)	555 (17.8)	531 (16.7)	
≥ RDA	1991 (22.6)	606 (20.5)	694 (23.7)	691 (23.5)	
**Mg supplements users, n(%)**	2890 (31.2)	1155 (36.1)	967 (31.3)	768 (26.1)	*< 0.001*
**Muscle-related medications, n (%)**	1813 (16.4)	676 (17.9)	601 (15.8)	536 (15.5)	*0.14*

^a^Categorical variables were presented as number (weighted percentage) and continuous variables were presented as mean ± standard deviation or median (IQR).

^b^ All estimates are weighted to be nationally representative

*Abbreviations* ASMI: appendicular skeletal muscle mass index; BMI: body mass index; CRP: C-reactive protein; EAR: estimated average requirement; Mg: magnesium; PIR: poverty income ratio; RDA: recommended dietary allowance

### Positive association of dietary mg intake with ASMI

**Fig. 2:**
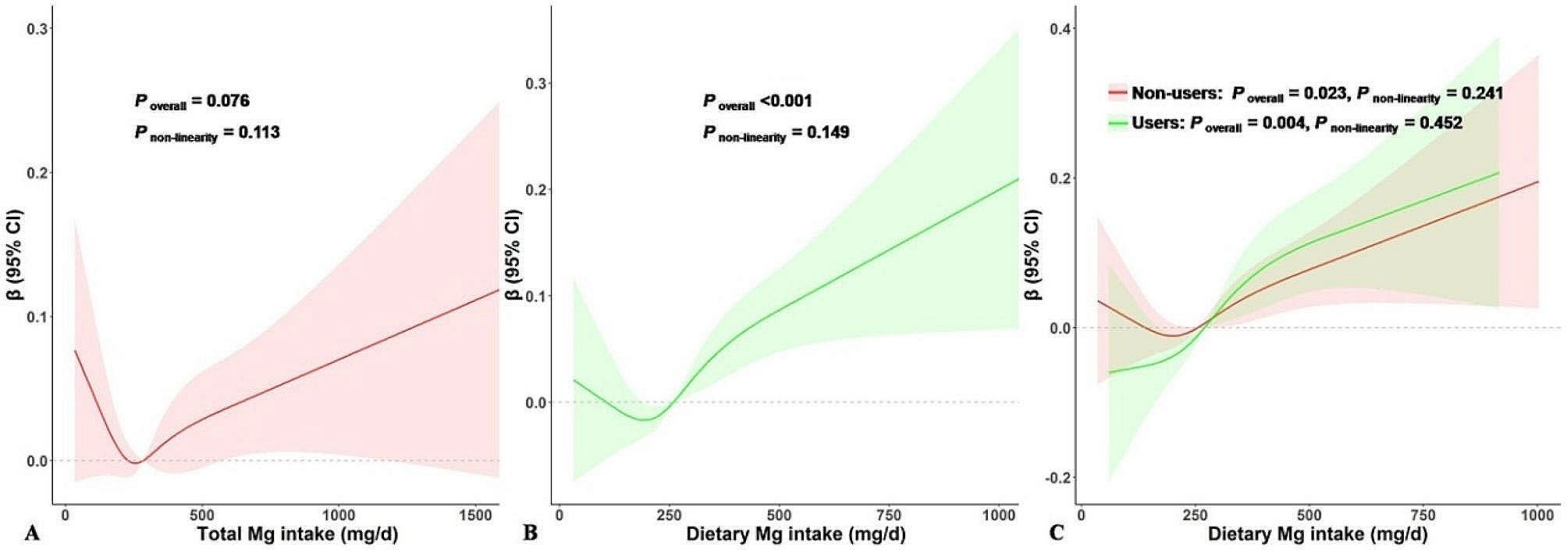
Dose-response relationship between Mg intake and ASMI in patients with hypertension. Panel A represents the association between total Mg intake and ASMI, panel B and C represent the association between dietary Mg intake and ASMI in all participants (B), and in users and nonusers of Mg supplements (C).Age as categorical variable (< 45, 45 ∼ 65, ≥ 65), gender, race, poverty income ratio, BMI, physical activity, smoking status, drinking status, dietary energy intake, dietary protein intake, total cholesterol, serum albumin, CRP, diabetic mellitus, heart disease, chronic kidney disease, and muscle-related medications were adjusted. Model for dietary Mg intake additionally adjusted for Mg intake from supplements. *Abbreviations* BMI, body mass index; CI, confidence interval; CRP: C-reactive protein; Mg, magnesium; mg/d, milligram per day

Results of dose-response analyses indicated a positive linear association between the total Mg intake and the ASMI (*P* for non-linearity = 0.113, Fig. 2A). Every additional 100 mg/day of total Mg intake was associated with 0.01 higher ASMI (95% confidence interval [CI]: 0.003–0.03) after adjustment. However, the associations became statistically insignificant after total Mg intake was categorized into < EAR (reference), EAR–<RDA, and ≥ RDA (*P* for trend = 0.137, Table 2).

**Table 2:** Association between Mg intake and muscle mass (*β*, 95%CI; *N* = 10,279)

	Model 1	Model 2	Model 3
Total Mg intake (from diet and supplements)			
Per 100 mg/day increment	0.08 (0.05, 0.11)^**^	0.02 (-0.002, 0.04)	0.01 (0.003, 0.03)
Categorical variable			
<EAR	Ref	Ref	Ref
EAR-<RDA	-0.06 (-0.20, 0.08)	0.03 (-0.09, 0.14)	0.04 (-0.01, 0.09)
≥RDA	-0.09 (-0.21, 0.03)	0.07 (-0.02, 0.16)	0.10 (-0.01, 0.10)
*P for trend*	*0.302*	*0.297*	*0.137*
**Dietary Mg intake**			
Per 100 mg/day increment	0.22 (0.19, 0.25)^**^	0.04 (0.02, 0.07) ^**^	0.04 (0.02, 0.06)^**^
Categorical variable			
<EAR	Ref	Ref	Ref
EAR-<RDA	0.08 (-0.06, 0.22)	0.15 (0.03, 0.26) ^**^	0.06 (0.01, 0.11) ^*^
≥RDA	0.14 (0.01, 0.26) ^*^	0.01 (0.01, 0.21) ^*^	0.10 (0.04, 0.16) ^**^
*P for trend*	*0.085*	*0.011*	*0.002*

Model 1: crude analysis

Model 2: adjusted for age as categorical variable (< 45, 45 ∼ 65, ≥ 65), gender, and race

Model 3: adjusted for age as categorical variable (< 45, 45 ∼ 65, ≥ 65), gender, race, poverty income ratio, BMI, physical activity, smoking status, drinking status, total energy intake, protein intake, diabetes, heart disease, chronic kidney disease, total cholesterol, serum albumin, CRP, and muscle-related medications. Model for dietary Mg intake was adjusted for Mg intake from supplements additionally

*Abbreviations* BMI: body mass index; CI: Confidence Interval; CRP: C-reactive protein; Mg: magnesium; EAR: estimated average requirement; RDA: recommended dietary allowance; Ref: reference

** *P* < 0.01; * *P* < 0.05

When sources of Mg intake were further evaluated, a positive linear correlation was found between dietary Mg intake and ASMI, irrespective of the use of Mg supplements (*P* for non-linearity > 0.05, Fig. 2B and C). In the fully adjusted model (Model 3), every additional 100 mg/day in dietary Mg was associated with 0.04 kg/m^2^ (95% CI: 0.02–0.06 kg/m^2^) higher ASMI. Participants in the EAR–<RDA group (*β* = 0.06, 95% CI: 0.01–0.11) and the ≥ RDA group (*β* = 0.10, 95% CI: 0.04–0.16) had a higher ASMI compared with those whose dietary Mg was below EAR (*P* for trend = 0.002, Table 2). Results were similar when these analyses were repeated in participants who met the criteria of SBP ≥ 140 mmHg and /or DBP ≥ 90 mmHg or in participants without hypertension history (Supplementary Tables S2, S3).

### Consistent associations between dietary mg intake and ASMI in different strata

Stratified analyses showed such positive associations between dietary Mg and ASMI were consistent across different strata of gender, age, PIR, BMI, physical activity, diabetes, heart disease, chronic kidney disease, energy intake, CRP, serum albumin, and the use of muscle-related medications (Table 3). Of note, protein intake had significant interactions with dietary Mg intake (*P* for interaction = 0.026). The association between dietary Mg intake and ASMI was stronger in participants without low protein intake (≥ 0.6 g/kg/d).

**Table 3:** Subgroup analysis for the association between dietary Mg intake and ASMI (*β*, 95%CI)

Exposure	Dietary Mg intake (mg/day)			
	<EAR	EAR-<RDA	≥RDA	P for interaction
Gender				*0.229*
Female	Ref	0.03 (-0.03, < 0.09)	0.14 (0.06, 0.21)	
Male	Ref	0.08 (0.00, 0.17)	0.11 (0.04, 0.19)	
Age (years)				*0.489*
< 65	Ref	0.07 (0.01, 0.13)	0.12 (0.06, 0.19)	
≥ 65	Ref	0.01(-0.10, 0.12)	0.04 (-0.06, 0.14)	
PIR				*0.486*
< 1	Ref	0.07 (-0.08, 0.22)	0.16 (0.02, 0.22)	
1 ≤ PIR < 3	Ref	0.02 (-0.08, 0.12)	0.10 (0.01, 0.18)	
≥ 3	Ref	0.08 (0.02, 0.15)	0.11 (0.03, 0.18)	
BMI (kg/m^2^)				*0.121*
< 30	Ref	0.10 (0.02, 0.17)	0.10 (0.04, 0.17)	
≥ 30	Ref	0.01 (-0.08, 0.09)	0.12 (0.03, 0.22)	
Physical activity				*0.376*
Less than moderate	Ref	0.02 (-0.07, 0.11)	0.11 (0.02, 0.19)	
Moderate	Ref	0.07(-0.02, 0.15)	0.10 (0.03, 0.18)	
Vigorous	Ref	0.11 (0.00, 0.21)	0.12 (0.00, 0.23)	
Diabetes				*0.142*
No	Ref	0.07 (0.02, 0.13)	0.10 (0.04, 0.16)	
Yes	Ref	-0.04 (-0.19, 0.10)	0.19 (0.04, 0.35)	
Heart disease				*0.480*
No	Ref	0.05 (0.00, 0.11)	0.11 (0.05, 0.17)	
Yes	Ref	0.13 (-0.03, 0.30)	0.11 (-0.08, 0.29)	
Chronic kidney disease				*0.490*
No	Ref	0.06 (0.00, 0.11)	0.11 (0.06, 0.17)	
Yes	Ref	0.08 (-0.06, 0.21)	0.09 (-0.07, 0.25)	
Low energy intake				*0.139*
No	Ref	0.09 (0.02, 0.17)	0.12 (0.06, 0.19)	
Yes	Ref	0.00 (-0.08, 0.07)	0.10 (0.00, 0.20)	
Low protein intake				***0.026***
No	Ref	0.07 (0.02, 0.12)	0.11 (0.06, 0.17)	
Yes	Ref	-0.11 (-0.31, 0.09)	-0.05 (-0.25, 0.16)	
CRP(mg/dL)^a^				*0.108*
< 0.35	Ref	0.03 (-0.05, 0.11)	0.09 (0.01, 0.17)	
≥ 0.35	Ref	0.07 (-0.01, 0.16)	0.10 (0.00, 0.20)	
Serum albumin (g/L)				*0.742*
< 38	Ref	-0.04 (-0.24, 0.16)	-0.18 (-0.39, 0.04)	
≥ 38	Ref	0.06 (0.01, 0.12)	0.12 (0.06, 0.17)	
Muscle-related medications				*0.265*
No	Ref	0.06 (0.00, 0.12)	0.11 (0.05, 0.12)	
Yes	Ref	0.05 (-0.08, 0.17)	0.07 (-0.06, 0.20)	

Each stratification was adjusted for age as categorical variable (< 45, 45 ∼ 65, ≥ 65), gender, race, PIR, BMI, physical activity, smoking status, drinking status, total energy intake, protein intake, diabetes, heart disease, chronic kidney disease, total cholesterol, serum albumin, CRP, and muscle-related medications except the stratification factor itself. Mg intake from supplements

^a^CRP is divided in two groups by median

*Abbreviations* BMI: body mass index; CI: confidence interval; CRP: C-reactive protein; EAR: estimated average requirement; Mg: magnesium; PIR: poverty income ratio; RDA: recommended dietary allowance; Ref: reference

### No association of mg supplements with ASMI

No association was observed between Mg supplements and ASMI. The ASMI in users of Mg supplements was similar to that in non-users (*β*=-0.04, 95% CI: -0.08–0.003; Table 4). When the analyses were limited to users of Mg supplements, neither continuous nor categorical Mg intake from supplements was related to ASMI, after adjustment for dietary Mg intake and other covariates.

**Table 4:** Association between Mg supplement use and muscle mass (*β*, 95%CI)

	Model 1	Model 2	Model 3
**Users versus non-users (N = 10,279)**	-0.38 (-0.49,-0.27)^**^	-0.09 (-0.18, -0.01)^*^	-0.04 (-0.08,0.003)
**Mg intake from supplements among users (N = 2890)**			
Per 100 mg/day increment	-0.04 (-0.08, -0.005)	0.00 (-0.03, 0.03)	0.01 (-0.01, 0.02)
Categorical variable (mg/day)			
Tertile 1 (≤ 50.00)	Ref	Ref	Ref
Tertile 2 (50.02–100.00)	-0.39 (-0.56, -0.22) ^**^	-0.13 (-0.28, 0.02)	-0.04 (-0.11, 0.03)
Tertile 3 (≥ 100.01)	-0.06 (-0.27, 0.15)	-0.02 (-0.19, 0.15)	0.03 (-0.06, 0.12)
*P for trend*	*< 0.001*	*0.212*	*0.189*

Model 1: crude analysis

Model 2: adjusted for age as categorical variable (< 45, 45 ∼ 65, ≥ 65), gender, and race

Model 3: adjusted forage as categorical variable (< 45, 45 ∼ 65, ≥ 65), gender, race, PIR, BMI, physical activity, smoking status, drinking status, total energy intake, protein intake, diabetes, heart disease, chronic kidney disease, total cholesterol, serum albumin, CRP, muscle-related medications, and dietary Mg intake

*Abbreviations* BMI: body mass index; CI: confidence interval; CRP: C-reactive protein; Mg: magnesium; PIR: poverty income ratio; Ref: reference

## Discussion

In this nationwide cross-sectional survey in 10,279 participants with hypertension based on NHANES, a positive association was observed between dietary Mg intake and ASMI, but not between Mg supplements and ASMI, implying the importance and uniqueness of dietary source of Mg. Adequate Mg intake from diet other than supplements may be a factor that prevents skeletal muscle loss in hypertensive patients. Similar results were found when the analyses were stratified by age, gender, BMI, physical activity, and comorbidities, suggesting that this association may apply to different population settings. To our knowledge, this is the first study to investigate the role of Mg intake in skeletal muscle loss among patients with hypertension.

Recently, some studies have found the relationship between hypertension and muscle mass [3, 31, 32]. A multicenter, cross-sectional study of 2,613 participants found that muscle mass was independently and negatively related to hypertension [31]. A study from Turkey reported that low muscle mass was more commonly seen in hypertensive (41.9%) when compared to normotensive adults (13.2%) [3]. The prevalence of low muscle mass in our study was 9.2%, which was lower than that in the Turkey study. This might be due to the fact that their participants were much older than ours (≥ 45 years vs. ≥ 20 years). Besides, different parts of the muscles were measured using different methods and different definitions of low muscle mass were applied.

Our findings elucidated a protective role of dietary Mg in maintaining muscle mass among hypertensive patients, which is in line with previous studies conducted in the general population [18–20, 33]. A systemic review about minerals and muscle mass suggested that Mg may regulate health through muscle activity [20]. Moreover, a study including 766 adolescents also found that lower Mg intake was associated with lower muscle mass [33]. Inadequate Mg intake is common in the U.S. population. About 22.3–42.8% of U.S. adults do not meet the EAR of daily Mg intake [34]. Our study found that this number is much higher in hypertensive patients (61%). Thus, enhancing public awareness of meeting the recommended level for Mg in hypertensive patients may help increase the ASMI and thus lower their odds of muscle loss.

The positive correlation between dietary Mg intake and ASMI could be explained by several possibilities. Animal studies have suggested that Mg might improve exercise performance by enhancing glucose availability in the muscle and blood [35]. Mg may also affect muscle performance though energy metabolism to maintain protein synthesis and turnover in the muscle [36]. In addition, recent studies have indicated that Mg deficiency might increase inflammatory response and associate with damage of muscle. Inflammation is one of the important factors involved in the occurrence and development of sarcopenia and hypertension [37, 38]. Experimental animals with Mg deficiency showed systemic inflammation with increased levels of inflammatory markers and dietary Mg supplementation reduced pro-inflammatory cytokine production and oxidative stress [39]. Several studies have indicated that higher Mg consumption was related to lower serum CRP [40].

In our study, no association was observed between Mg supplements and muscle mass, which is consistent with the results of previous randomized controlled studies [41, 42], implying that whole grains and nuts rich in high amounts of natural Mg were recommended instead of dietary supplements. These foods may exert additional benefit since they also contain other nutrients (vitamin D, vitamin B6, etc.) that can directly increase muscle mass or indirectly do so by increasing the bioavailability of Mg or by reducing the risk of hypertension, a risk factor for muscle loss [43, 44]. Different bioavailability of Mg from diet and supplements may also partly explain the results [14, 45].

Intriguingly, the positive correlation between dietary Mg intake and ASMI was higher in participants without low protein intake (protein intake ≥ 0.6 g/kg/d). Inadequate protein intake has been shown to lead to muscle loss besides other predisposing factors [29, 30]. It may mask the association between Mg and muscle mass. What’s more, protein intake may influence the bioavailability of Mg. Some studies showed that higher protein intake increased Mg absorption compared to lower intake [46, 47]. Both low protein intake and inadequate Mg intake need to be modified to preserve muscle mass.

There are some limitations to this study. First, cross-sectional research only allows us to investigate the correlation between dietary Mg intake and skeletal muscle mass instead of a causal relationship. Second, Mg intake was based on self-report and therefore was subject to recall bias. However, ingredient and dosage information were obtained from the bottles and nutrition fact labels during home examinations, which may reduce the bias. Third, data on muscle strength and function were not available in this study, so the relationships between these variables and dietary Mg could not be investigated. Thus, further prospective studies involving the association between muscle function and dietary Mg intake are needed. Fourth, the participants who had a history of hypertension might change their dietary pattern. To minimize the influence of bias, we performed a sensitivity analysis in participants who did not have hypertension history or were taking medications for hypertension. The results remained stable.

Nevertheless, this is the first time to investigate the association between Mg intake and muscle mass among patients with hypertension based on the public NHANES database, and both Mg from diet and from supplements are taken into consideration. The large and non-institutionalized samples make our results more convincing and applicable. Besides, no profound changes were observed in the stratified and sensitivity analyses, suggesting that our results were robust. Moreover, the comprehensive data from NHANES allowed us to adjust for a wide range of confounders such as lifestyle factors, socioeconomic status, ethnicity, and comorbidities.

## Conclusions

In conclusion, there was a positive linear correlation between dietary Mg intake and skeletal muscle mass in the hypertensive population. We should advocate sufficient Mg intake from diet for patients with hypertension to achieve better prevention of muscle loss. Longitudinal studies and clinical trials are needed to assess the causal relationship in the future.

### Electronic supplementary material

Below is the link to the electronic supplementary material.

Supplementary Material 1

## Data Availability

The NHANES database is publicly available at the NHANES website (https://www.cdc.gov/nchs/nhanes/index.htm).
