# Supplementary material for: Association between dietary magnesium intake and muscle mass among hypertensive population: evidence from the National Health and Nutrition Examination Survey
Source: Nutr J. 2024 Mar 21;23:37. doi: 10.1186/s12937-024-00940-6 (PMC10956219; doi:10.1186/s12937-024-00940-6)
Supplement: Supplementary file 1 — Supplementary Material 1 [file 12937_2024_940_MOESM1_ESM.docx]

Table S1.the estimated average requirement (EAR) and recommended dietary allowance (RDA) for dietary magnesium

|  | Age (years) | |
| --- | --- | --- |
|  | 19~30 | >31 |
| EAR for dietary Mg (mg/d) | | |
| female | 255 | 265 |
| male | 330 | 350 |
| RDA for dietary Mg (mg/d) | | |
| female | 310 | 320 |
| male | 400 | 420 |

Table S2. Association (β, 95%CI) between dietary magnesium intake and ASMI among participants with blood pressure over 140/90 mmHg (n=7429)

|  | **Model 1** | **Model 2** | **Model 3** |
| --- | --- | --- | --- |
| Continuous variable (per 100mg/day increment) | | | |
| Dietary magnesium | 0.23 (0.19-0.28)** | 0.05 (0.01-0.08)** | 0.04 (0.02-0.07)** |
| Categorical variable |  |  |  |
| Quartile 1 | Ref | Ref | Ref |
| Quartile 2 | 0.22 (0.06-0.38)** | 0.03 (-0.09-0.16) | 0.03 (-0.02-0.08) |
| Quartile 3 | 0.42 (0.26-0.58)** | 0.12 (0.00-0.25) | 0.04 (-0.03-0.10) |
| Quartile 4 | 0.85 (0.69-1.00)** | 0.19 (0.06-0.32)** | 0.13 (0.05-0.22)** |
| P for trend | <0.001 | 0.012 | 0.013 |
| Categorical variable |  |  |  |
| <EAR | Ref | Ref | Ref |
| EAR-RDA | 0.12 (-0.04-0.28) | 0.19 (0.05-0.33)** | 0.05 (-0.01-0.12) |
| ≥RDA | 0.17 (0.03-0.31)* | 0.12 (0.00-0.23)* | 0.10 (0.03-0.17)** |
| P for trend | 0.052 | 0.017 | 0.015 |

Model 1: not adjusted

Model 2: adjusted by age, gender, and race

Model 3: adjusted by age, gender, race, PIR, BMI, physical activity, smoking status, drinking status, DM, dietary energy intake, dietary protein intake, use of dietary supplements, WBC, serum albumin, TC, DM, heart disease, CKD and use of muscle loss related drugs

Dietary Mg group: Quartile 1: 9.0-184.92 mg/day, Quartile 2: 185.0-252.87 mg/day, Quartile 3: 253.0-340.0 mg/day, Quartile 4: 340.03-1714.0 mg/day; <EAR: 9.0-349.5 mg/day, EAR-RDA: 255.0-420.0 mg/day, ≥RDA:312.5-1714.0 mg/day

Abbreviations: ASMI: appendicular skeletal muscle mass index; CI: Confidence Interval; Ref: reference; Mg: magnesium; EAR: estimated average requirement; RDA: recommended dietary allowance; OR: odds ratio

**P < 0.01; *P < 0.05.

Table S3. Association (β, 95%CI) between dietary magnesium intake and ASMI among hypertensive participants without hypertension history (n=4396)

|  | **Model 1** | **Model 2** | **Model 3** |
| --- | --- | --- | --- |
| Continuous variable (per 100mg/day increment) | | | |
| Dietary magnesium | 0.21 (0.17-0.25)** | 0.04 (0.00-0.07)* | 0.05 (0.03-0.08)** |
| Categorical variable |  |  |  |
| Quartile 1 | Ref | Ref | Ref |
| Quartile 2 | 0.21 (-0.02-0.45) | 0.03 (-0.13-0.20) | 0.04 (-0.03-0.11) |
| Quartile 3 | 0.45 (0.24-0.67)** | 0.02 (-0.15-0.20) | 0.09 (0.01-0.17)* |
| Quartile 4 | 0.83 (0.64-1.02)** | 0.14 (-0.02-0.29) | 0.17 (0.07-0.26)** |
| P for trend | <0.001 | <0.239 | 0.003 |
| Categorical variable |  |  |  |
| <EAR | Ref | Ref | Ref |
| EAR-RDA | 0.10 (-0.08-0.28) | 0.13(-0.01-0.26) | 0.11 (0.05-0.17)* * |
| ≥RDA | 0.11 (-0.09-0.31) | 0.08 (-0.07-0.23) | 0.13 (0.05-0.21)** |
| P for trend | 0.383 | 0.123 | <0.001 |

Model 1: not adjusted

Model 2: adjusted by age, gender, and race

Model 3: adjusted by age, gender, race, PIR, BMI, physical activity, smoking status, drinking status, DM, dietary energy intake, dietary protein intake, use of dietary supplements, WBC, serum albumin, TC, DM, heart disease, CKD and use of muscle loss related drugs

Dietary Mg group: Quartile 1:11.0-188.0 mg/day, Quartile 2: 188.5-259.0 mg/day, Quartile 3: 259.5-348.6 mg/day, Quartile 4: 349.0-1654.0 mg/day; <EAR: 11.0-349.5 mg/day, EAR-RDA: 255.0-420.0 mg/day, ≥RDA:314.0-1654.0 mg/day

Abbreviations: ASMI: appendicular skeletal muscle mass index; CI: Confidence Interval; Ref: reference; Mg: magnesium; EAR: estimated average requirement; RDA: recommended dietary allowance; OR: odds ratio

**P < 0.01; *P < 0.05.

Table S4. Association (β, 95%CI) between total dietary magnesium intake and ASMI among hypertensive participantswith magnesium data of both dietary and dietary supplements (NHANES 2011-2018, n=3470)

|  | **Model 1** | **Model 2** | **Model 3** |
| --- | --- | --- | --- |
| Continuous variable (per 100mg/day increment) | | | |
| Dietary magnesium | 0.17 (0.12-0.22)** | 0.06 (0.02-0.11)** | 0.04 (0.01-0.08)** |
| Categorical variable |  |  |  |
| Quartile 1 | Ref | Ref | Ref |
| Quartile 2 | 0.11 (-0.15-0.36) | -0.02 (-0.22-0.18) | 0.06 (-0.02-0.14) |
| Quartile 3 | 0.37 (0.11-0.62)** | 0.12 (-0.08-0.31) | 0.10 (-0.00-0.21) |
| Quartile 4 | 0.65 (0.41-0.90)** | 0.18 (-0.02-0.38) | 0.16 (0.03-0.30)* |
| P for trend | <0.001 | 0.12 | 0.10 |
| Categorical variable |  |  |  |
| <EAR | Ref | Ref | Ref |
| EAR-RDA | 0.04 (-0.24-0.32) | 0.12 (-0.11-0.35) | 0.09 (-0.01-0.19) |
| ≥RDA | 0.04 (-0.06-0.256) | 0.15 (-0.01-0.30) | 0.12 (0.02-0.23)* |
| 0.910 | 0.910 | 0.168 | 0.034 |

Model 1: not adjusted

Model 2: adjusted by age, gender, and race

Model 3: adjusted by age, gender, race, PIR, BMI, physical activity, smoking status, drinking status, DM, dietary energy intake, dietary protein intake, use of dietary supplements, WBC, serum albumin, TC, DM, heart disease, CKD and use of muscle loss related drugs

Dietary Mg group: Quartile 1: 9.5-212.0 mg/day, Quartile 2: 212.5-291.0 mg/day, Quartile 3: 291.5-393.0 mg/day, Quartile 4: 393.5-1714.0 mg/day; <EAR: 9.5-349.5 mg/day, EAR-RDA: 255.0-419.5 mg/day, ≥RDA:312.5-1714.0 mg/day

Abbreviations: ASMI: appendicular skeletal muscle mass index; CI: Confidence Interval; Ref: reference; Mg: magnesium; EAR: estimated average requirement; RDA: recommended dietary allowance; OR: odds ratio

**P < 0.01; *P < 0.05.
